# Using reaction times and binary responses to estimate psychophysical performance: an information theoretic analysis

**DOI:** 10.3389/fnins.2014.00035

**Published:** 2014-03-04

**Authors:** James V. Stone

**Affiliations:** Psychology Department, Sheffield UniversitySheffield, UK

**Keywords:** psychometric function, chronometric function, point of subjective equality, diffusion model, reaction time, threshold, Shannon information, mutual information

## Abstract

As the strength of a stimulus increases, the proportions of correct binary responses increases, which define the psychometric function. Simultaneously, mean reaction times (RT) decrease, which collectively define the chronometric function. However, RTs are traditionally ignored when estimating psychophysical parameters, even though they may provide additional Shannon information. Here, we extend Palmer et al's (2005) proportional-rate diffusion model (PRD) by: (a) fitting individual RTs to an inverse Gaussian distribution, (b) including lapse rate, (c) point-of-subjective-equality (PSE) parameters, and, (d) using a two-alternative forced choice (2AFC) design based on the proportion of times a variable comparison stimulus is chosen. Maximum likelihood estimates of mean RT values (from fitted inverse Gaussians) and binary responses were fitted both separately and in combination to this extended PRD (EPRD) model, to obtain psychophysical parameter values. Values estimated from binary responses alone (i.e., the psychometric function) were found to be similar to those estimated from RTs alone (i.e., the chronometric function), which provides support for the underlying diffusion model. The EPRD model was then used to estimate the mutual information between binary responses and stimulus strength, and between RTs and stimulus strength. These provide conservative bounds for the average amount of Shannon information the observer gains about stimulus strength on each trial. For the human experiment reported here, the observer gains between 2.68 and 3.55 bits/trial. These bounds are monotonically related to a new measure, the *Shannon increment*, which is the expected value of the smallest change in stimulus strength detectable by an observer.

## 1. Introduction

For over a 100 years, it has been known that the ability to discriminate between two stimuli increases as a sigmoidal function of the difference between those stimuli, where this is traditionally measured using binary observer responses. However, when an observer makes a response, there is a trade-off between speed, or reaction time (RT), and accuracy of responses. This speed-accuracy trade-off has been the subject of numerous papers, notably (Ratcliff, 1978; Harvey, 1986; Swanson and Birch, 1992; Wichmann and Hill, 2001; Palmer et al., 2005), and more recently in Bonnet et al. (2008).

Here, we propose four extensions to the proportional-rate diffusion model (PRD) proposed in Palmer et al. (2005). First, we introduce a new parameter, the point-of-subjective-equality (PSE), which takes account of systematic shifts or bias in observer perception. This parameter is incorporated into the chronometric and psychometric functions. Second, we use a maximum likelihood estimate (MLE) of the RT mean based on a physically motivated diffusion model of RTs which involves fitting individual RTs to an inverse Gaussian distribution. Third, we take account of lapses in observer concentration by introducing a lapse rate parameter, which is estimated simultaneously with other psychophysical parameters. Fourth, we use a two-alternative forced choice (2AFC) design where the psychometric function is defined, not by the proportion of correct responses (range 50–100%), but by the proportion of times a variable comparison stimulus is chosen in preference to a fixed reference stimulus (range 0–100%). Note that the 2AFC experimental procedure is the same whether one chooses to measure the proportion of correct responses or the proportion of times a variable comparison stimulus is chosen.

Once the model has been fitted to these data, it can be used to estimate the mutual information (Shannon and Weaver, 1949; MacKay, 2003; Stone, 2014) between binary responses and stimulus strength, and between RT and stimulus strength. Finally, the mutual information provides a value for the Shannon increment, which is the expected value of the smallest change in stimulus strength detectable by an observer.

## 2. The proportional-rate diffusion model

We provide a brief summary of Palmer et al's PRD model (Palmer et al., 2005) here, and describe extensions below. In the experiment described in Palmer et al. (2005), an observer is presented with an array of moving dots. Stimulus strength *x* is defined by coherence (i.e., the percentage of dots moving in the same direction), and the observer is required to indicate which one of two directions the dots are moving. Note that coherence, and therefore stimulus strength *x*, varies between zero and some upper bound.

The PRD model is based on a diffusion model of RT, where the *mean* RT τ_PRD_ varies as a sigmoidal function of *x*

(1) $${\bar{\tau }}_{\text{PRD}}=\frac{A}{Kx}\tanh(KAx)+{\bar{\tau }}_{\text{res}},$$

where *K* is a measure of observer sensitivity, and *A* represents a decision boundary associated with RT. The first term on the right hand side represents the time to make a decision, and τ_res_ is a fixed residual RT (e.g., time to respond after a decision is made). Notice that this model requires that the mean RT τ_PRD_ decreases monotonically as the motion signal increases above zero, a requirement which will be relaxed in the model proposed below.

Within the PRD model, the probability *P*_PRD_ of making a correct response is defined by the logistic psychometric function

(2) $$P_{\text{PRD}}=\frac{1}{1+e^{-2AK|x|}},$$

where |*x*| indicates the absolute value of *x*. In Equation (2), the product *AK* acts as a single parameter which modulates the steepness of the sigmoidal function, and therefore acts as a measure of sensitivity to changes in stimulus strength. Note that the stimulus strength cannot fall below zero in Palmer et al's moving dot experiment, and that, when the stimulus motion strength is *x* = 0%, the observer has to guess, so that *P*_PRD_ = 0.5, whereas if *x* = 100% then *P*_PRD_ = 1.0.

## 3. The extended proportional-rate diffusion (EPRD) model

The model proposed here is based on the assumption that responses arise from a two-alternative forced choice (2AFC) procedure. On each trial, the observer is presented with two stimuli, and the task is to choose the stronger stimulus, where strength can be defined in terms of differences in any physical quantity, such as speed, luminance, or contrast. The two stimuli are a *reference stimulus* with a stimulus value *s*_*R*_ that remains constant within a specific subset of trials, and a *comparison stimulus* with a value *s*_*C*_ that varies between trials. A *comparison* response is obtained if the observer chooses the comparison stimulus. The stimulus strength *x* within one trial is defined as the difference between the reference value *s*_*R*_ and the comparison value *s*_*C*_, specifically *x* = *s*_*C*_ − *s*_*R*_.

We measure performance in terms of the proportion *P* of times that a variable comparison stimulus is chosen in preference to the fixed reference stimulus, which we define as a comparison stimulus response, so *P* varies between zero and one. A direct translation from *P*_PRD_ to *P* would guarantee that a stimulus strength of zero corresponds to *P* = 0.5. However, if observer perception is biased, such that a stimulus difference of *x* = 0 is not perceived as zero, then a stimulus strength of zero would not coincide with *P* = 0.5. This perceptual bias can be accommodated with a second modification, a new parameter *s*_PSE_, which is the point-of-subjective-equality (PSE) between the comparison and reference stimuli. Specifically, *s*_PSE_ is the value *s*_*C*_ of the comparison stimulus which is perceived to be the same as the value *s*_*R*_ of reference stimulus.

Given that the stimulus strength is *x* = *s*_*C*_ − *s*_*R*_, the *perceived stimulus strength x*′ is

(3) $$x^{\prime }=s_{C}-s_{\text{PSE}}$$

(4)    =x−Δx,

where Δ*x* is the error in the perceived value of *s*_*C*_. The probability of choosing the comparison stimulus is defined as

(5) $$P=\frac{1}{1+e^{-2AKx^{\prime }}}.$$

Note that the product *AK* effectively acts as a single parameter, and will be treated as such for binary response data (but not for RT data, see below).

In order to take account of observer lapses in concentration, which result in a pure guess, we introduce a lapse rate parameter γ. Evidence presented in Wichmann and Hill (2001) suggests that failure to take account of the lapse rate can lead to substantial errors in estimated psychophysical parameter values. If the lapse rate were zero then we would expect that *P* = 0 for highly negative stimulus strengths, and that *P* = 1 for highly positive stimulus strengths, so that observed deviations from *P* = 0 and *P* = 1 at extreme stimulus strengths can be used to provide an estimate of the lapse rate. Thus, the lapse rate parameter limits the lower and upper bounds of the psychometric function to *P*_min_ = γ/2 and *P*_max_ = 1 − γ/2, respectively, such that^1^

(6) $$P=\left[\frac{1}{1+e^{-2AKx^{\prime }}}-0.5\right](1-\gamma )+0.5.$$

Thus, the three parameters to be estimated for Equation (6) define the vector variable

(7) $$\theta_{P}=(s_{\text{PSE}},AK,\gamma ).$$

Similarly, we model the observer's mean RT for a perceived stimulus strength *x*′ as

(8) $$\bar{\tau }=\frac{A}{kx^{\prime }}\tanh\;(KAx^{\prime })+{\bar{\tau }}_{\text{res}}.$$

Here, the effects of *A* and *K* are separable, and so the four parameters to be estimated for Equation (8) define the vector variable

(9) $$\theta_{\tau }=(s_{\text{PSE}},A,K,{\bar{\tau }}_{\text{res}}).$$

The lapse rate parameter is not included here because lapses have no predictable effect on RT.

Finally, we can adapt results from Luce (1986) and Palmer et al. (2005) to relate RT to response probability. The mean decision time is defined as τ_dec_ = τ_*i*_ − τ_res_, so that Equations (5, 8) can be combined to provide a mapping between mean decision time τ_dec_ and the probability *P* of choosing the comparison stimulus

(10) $${\bar{\tau }}_{\text{dec}}=(A/K)\frac{\left(2P-1\right)}{x^{\prime }}.$$

Thus, if the perceived stimulus strength *x*′ has a large positive or negative value then *P* = 0 or *P* = 1 (respectively), and so τ_dec_ = *A*/(*K*|*x*′|) in both cases. This predicts that, for a given perceived stimulus strength, the probability of choosing the comparison stimulus is proportional to the mean decision time.

## 4. Using observer responses

For each trial, we obtain a RT and a binary response from the observer, which indicates whether the observer has chosen the comparison stimulus or the reference stimulus. At each stimulus strength *x*_*i*_, the comparison and reference stimuli are presented to the observer on *N*_*i*_ trials, and the number of times the observer chooses the comparison and reference stimulus is recorded as *n*_*i*_ and *N*_*i*_ − *n*_*i*_, respectively. For a given putative value of *P*_*i*_, a standard binomial model gives the probability of the observed binary responses as

(11) $$p(n_{i}|N_{i},P_{i})=C_{N_{i}}^{n_{i}}\times P_{i}^{n_{i}}\times {\left(1-P_{i}\right)}^{N_{i}-n_{i}},$$

where *P*_*i*_ is a function of the parameters *Ak*, γ and PSE as defined in Equation (6). The maximum likelihood estimate of *P*_*i*_ is the proportion of comparison stimulus responses *P*′_*i*_ = *n*_*i*_/*N*_*i*_.

When considered over all *N*_*x*_ values of *x*, the probability of observing the set of all binary responses is defined by the log likelihood function

(12) $$L_{P}=\log\prod_{i=1}^{N_{x}}C_{N_{i}}^{n_{i}}\;P_{i}^{n_{i}}{\left(1-P_{i}\right)}^{N_{i}-n_{i}}$$

(13) $$\;=\sum_{i=1}^{N_{x}}n_{i}\log P_{i}+\sum_{i=1}^{N_{x}}\left(N_{i}-n_{i}\right)\log(1-P_{i})+\sum_{i=1}^{N_{x}}\log C_{N_{i}}^{n_{i}},\;$$

where the final term does not depend on parameter values, and can be discarded unless the exact value of the likelihood is required. Recall that each *P*_*i*_ is determined by Equation (6), which is a function of the EPRD parameter values θ_*P*_ = (*A, K*, γ, PSE). The maximum likelihood estimate (MLE) of θ_*P*_ is obtained by finding EPRD parameter values θ_*P*_ that maximize *L*_*P*_.

If the number of trials at each stimulus strength is large then Equation (13) can be approximated by a Gaussian function. At a given stimulus strength *x*_*i*_, the observed proportion of binary responses is *P*′_*i*_, which is assumed to be the probability *P*_*i*_ plus a noise term η_*P*_, so that *P*′_*i*_ = *P*_*i*_ + η_*P*_. If the noise η_*P*_ has a Gaussian distribution with variance *v*_*P,i*_ then

(14) $$p({P^{\prime }}_{i}|A,k,{x^{\prime }}_{i})=\frac{1}{\sqrt{2\pi v_{P,i}}}\exp\frac{-{\left({P^{\prime }}_{i}-P_{i}\right)}^{2}}{2v_{P,i}},$$

where *P*_*i*_ is defined as a function of *A, k, x*′ in Equation (6), and the variances *v*_*P,i*_ can be estimated from the data as *v*_*P,i*_ = *N*_*i*_*P*′_*i*_(1 − *P*′_*i*_). Results for the Gaussian approximation in Equation (14) were found to be very similar to those for Equation (13). Results reported here are based on Equation (13).

## 5. Using reaction times

RTs tend to be short if the comparison stimulus value is very different from the reference stimulus, but as the comparison and reference stimuli become more similar, so the RT increases, as shown in Figure 4B. Here, we use RTs in a two stage process. First, a mean RT value is estimated at each stimulus strength. These mean RT values are then used as data for the *RT*_τ_ model, which is used to estimate EPRD model parameters.

### 5.1. Inverse gaussian model of individual RTs

It is commonly assumed that the RT is the time required for the cumulative amount of perceptual evidence to reach some criterion value (Ratcliff, 1978; Smith, 1990). Specifically, this evidence accumulation is assumed to consist of a Brownian diffusion process with positive drift, which can be likened to a the total distance traveled in a one-dimensional biased random walk. If a Brownian process is allowed to run for a *fixed time* then it is well known that the final distribution of values (e.g., evidence) has a Gaussian distribution. However, it is less well known that if a Brownian diffusion process is allowed to run until it reaches a *fixed criterion value* then the time taken to reach that value has an *inverse Gaussian* or *Wald* distribution (see Figure 3). Therefore, if the amount of evidence required to make a response is stable for a given observer then RTs are appropriately modeled using an inverse Gaussian distribution^2^.

**Figure 1:**
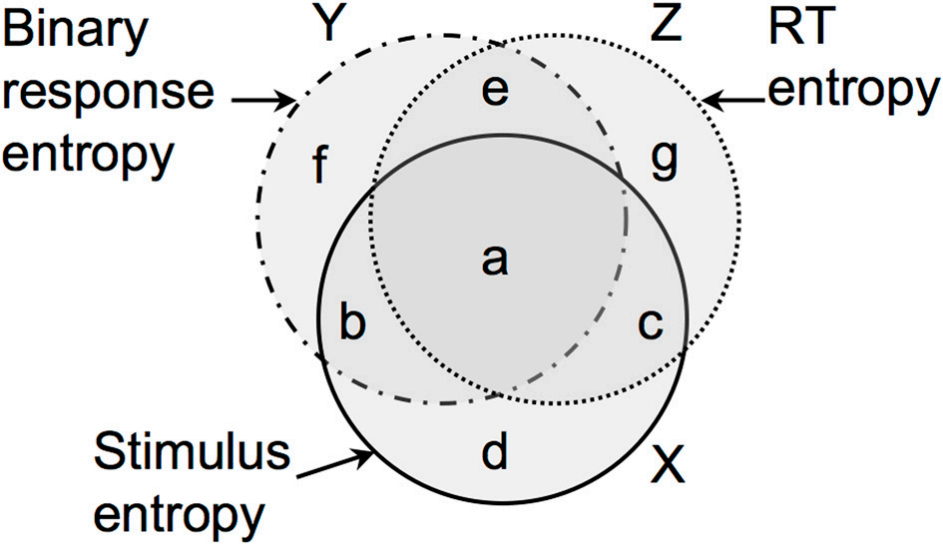
**How the entropy** *H*(*x*) **in stimulus strength** *x* **is accounted for by the entropy** *H*(τ) **in RT** (τ) **and entropy** *H*(*P*) **in the probability** *P* **of a particular binary response** *r*. The entropies of *x, P*, and τ are represented by the discs *X, Y*, and *Z*, respectively. The mutual information between *x* and *P* is *I*(*x, P*) = (*a* + *b*), and the mutual information between *x* and τ is *I*(*x*, τ) = (*a* + *c*).

**Figure 2:**
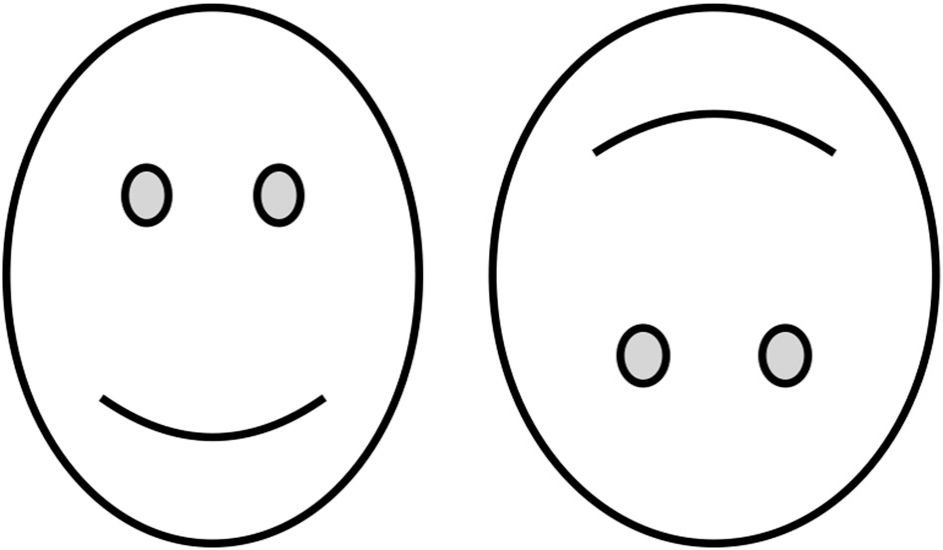
**Schematic illustration of typical stimulus shown to observer on a single trial.** The observer has to choose the face that looks wider. The stimulus in the experiment used was a picture of the actor James Corden's face, with all background details removed (see http://illusionoftheyear.com/2010/the-fat-face-thin-fft-illusion).

**Figure 3:**
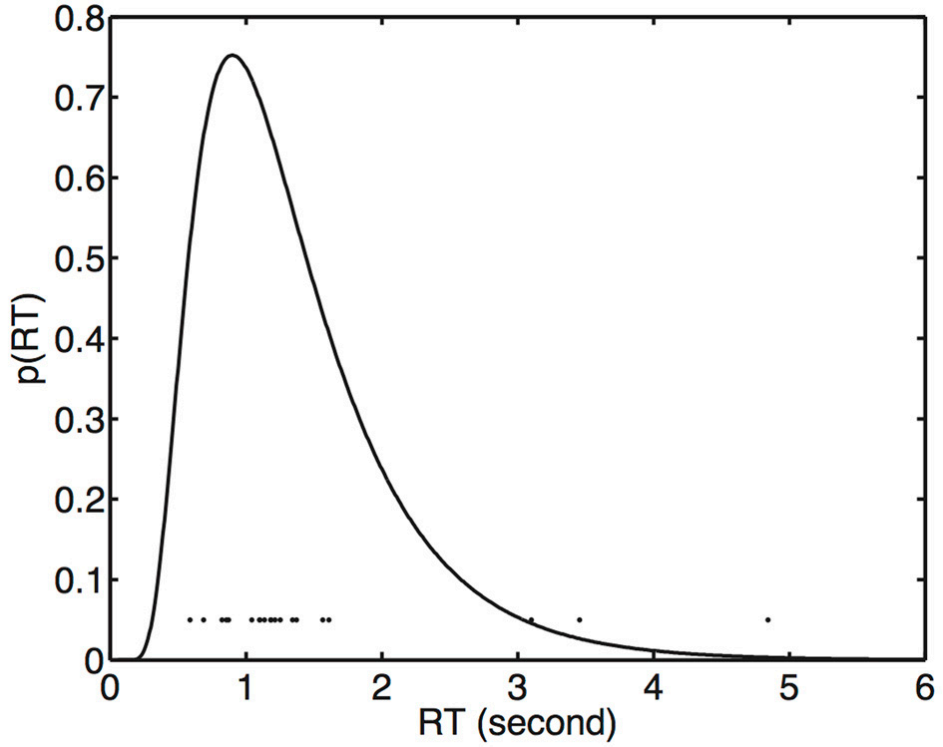
**Reaction times fitted with an inverse Gaussian (Equation 15).** Each dot represents 1 of 20 RTs for a stimulus value (width scaling) of *s* = 1.05.

If RTs have an inverse Gaussian distribution with mean τ′_*i*_ then the probability of a single observed RT τ_*ij*_ associated with the *j*th presentation of the stimulus value *x*_*i*_ is

(15) $$p(\tau_{ij}|{{\bar{\tau }}^{\prime }}_{i},\lambda_{i})={\left(\frac{\lambda_{i}}{2\;\pi \;\tau_{ij}^{3}}\right)}^{1/2}\times \exp\left[\frac{-\lambda_{i}{\left(\tau_{ij}-{{\bar{\tau }}^{\prime }}_{i}\right)}^{2}}{2\;{{\bar{\tau }}^{\prime }}_{i}{}^{2}\;\tau_{ij}}\right]\text{​},$$

where the variance of this distribution is

(16) $$v_{\tau_{i}}={{\bar{\tau }}^{\prime }}_{i}^{3}3/\lambda_{i}.$$

Each of the *N*_*x*_ stimulus strengths is presented *N*_*i*_ times. For one model RT mean, the probability of the observed *N*_*i*_ RTs (one RT per trial) defines the log likelihood function

(17) $$L_{\tau ,i}=\log\prod_{j=1}^{N_{i}}p(\tau_{ij}|{{\bar{\tau }}^{\prime }}_{i},\lambda_{i}).$$

Maximizing Equation (17) with respect to the parameters τ′_*i*_ and λ_*i*_ yields a maximum likelihood estimate (MLE) of both parameters at one stimulus strength *x*_*i*_. Even though the algebraic mean and the MLE mean are identical (Tweedie, 1957) for the inverse Gaussian, the fitting process provides the parameter estimate λ_*i*_, which is vital for subsequent calculations.

### 5.2. Model *RT*_τ_: using mean reaction times

For a given stimulus strength *x*_*i*_, the predicted mean RT τ_*i*_ varies as a tanh function of *x*_*i*_, as defined in Equation (8). The central limit theorem allows us to assume that the distribution of mean RTs of the inverse Gaussian pdf at a given stimulus strength *x*_*i*_ is Gaussian with mean τ′_*i*_ and variance *v*_τ,*i*_. Therefore, the likelihood of the EPRD mean τ_*i*_ from Equation (8) is

(18) $$p({{\bar{\tau }}^{\prime }}_{i}|{\bar{\tau }}_{i}(\theta_{\tau }))=\frac{1}{\sqrt{2\pi v_{\bar{\tau },i}}}e^{-{\left({{\bar{\tau }}^{\prime }}_{i}-{\bar{\tau }}_{i}\right)}^{2}/(2v_{\bar{\tau },i})}.$$

The variance of an inverse Gaussian distribution of RT values with mean τ′_*i*_ is *v*_τ*i*_ (Equation 16), so the variance *v*_τ*i*_ of a distribution of means (where each mean is based on *N*_*i*_ samples) is

(19) $$v_{\bar{\tau }i}=\frac{{\bar{\tau }}_{i}^{\prime \;3}}{\lambda_{i}\;N_{i}}.$$

Thus, we can assess the fit of the inverse Gaussian mean RTs τ′_*i*_ to the EPRD mean RTs τ_*i*_ of Equation (8) as follows. The probability of the *N*_*x*_ mean RTs τ′_*i*_ (one mean RT per stimulus strength) defines the log likelihood function

(20) $$L_{\bar{\tau }}=\log\prod_{i=1}^{N_{x}}p({{\bar{\tau }}^{\prime }}_{i}|{\bar{\tau }}_{i})$$

(21) $$\;=-1/2\sum_{i=1}^{N_{x}}\frac{{\left({{\bar{\tau }}^{\prime }}_{i}-{\bar{\tau }}_{i}\right)}^{2}}{v_{\bar{\tau },i}}-1/2\sum_{i=1}^{N_{x}}\log2\pi v_{\bar{\tau },i},$$

where τ_*i*_ is defined in Equation (8), so that the parameters to be estimated for model *RT*_τ_ are θ_τ_ = (*A, k*, γ, PSE, τ_res_) to fit the overall variation in mean RT with stimulus strength *x*.

**Figure 4:**
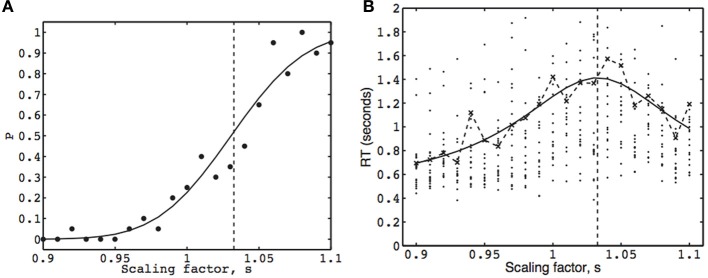
**The psychometric function (A) and chronometric function (B), from the face inversion experiment for one observer.** The width scaling factor *s* applied to the comparison image is indicated on the abscissa. The vertical dashed line marks the point-of-subjective-equality (PSE) at *s* = 1.032. **(A)** Each dot represents the observed proportion of trials for which the observer chose the comparison stimulus, and the fitted psychometric function is defined in Equation 6. **(B)** Each dot represents the RT of a single trial for the same responses as in Figure 4A (RTs greater than 2 s are not shown). The fitted chronometric function is defined in Equation 8. The dashed curve joins the fitted (inverse Gaussian) mean RTs, each of which was obtained by maximizing Equation 17. The solid curves in **(A, B)** (Equations 6, 8, respectively) were fitted using combined binary and mean RT data by maximizing Equation 22. A graph similar to **(A)** was obtained for model *L*_*P*_ (i.e., using only binary responses), and a graph similar to **(B)** was obtained for model *L*_τ_ (i.e., using only mean RTs).

In summary, we have three estimates of the mean RT at each stimulus strength: the algebraic mean τ′_obs*i*_, the MLE mean of the inverse Gaussian or Wald pdf τ′_*i*_ (from Equation 17), which collectively are used as data to estimate the means τ_*i*_ (one per stimulus strength) obtained from the fitted EPRD model (from Equation 21). The MLE means τ′_*i*_ are shown as crosses in Figure 4B, and the means τ_*i*_ are corresponding points on the fitted curve, respectively.

We also have two estimates of the probability of a comparison stimulus response at each stimulus strength: the observed proportion of comparison stimulus responses (which is the MLE *P*′_*i*_ = *n*_*i*_/*N*_*i*_), and the mean *P*_*i*_ (one per stimulus strength) obtained from fitting the EPRD model (Equation 13) to the MLE means *P*′_*i*_. These are shown as dots in Figure 4A, and as corresponding points on the fitted curve, respectively.

## 6. Using binary responses and RTs

In the absence of knowledge regarding the covariance between the noise in mean RT and binary response probability, we are forced to assume this covariance is zero. In other words, we assume that *L*_*P*_ and *L*_τ_ provide independent estimates of the EPRD model parameters. In this case, estimates based on combined RT and binary response probability are obtained by maximizing the sum of likelihoods

(22) $$L_{C}=L_{P}+L_{\bar{\tau }}.$$

However, the implausibility of this independence assumption means that we will not take seriously any results based on Equation (22).

## 7. Information theory

The amount of Shannon information (Shannon and Weaver, 1949; MacKay, 2003; Stone, 2014) that the observer gains about the stimulus is reflected in both the binary responses and RTs. Specifically, the average Shannon information that each mean RT provides about the stimulus strength *x* is the mutual information *I*(*x*, τ) between *x* and the mean RT. Similarly, the average Shannon information that binary responses provide about the stimulus strength *x* is the mutual information *I*(*x,P*) between *x* and the probability of a comparison stimulus binary response.

More importantly, the total amount of Shannon information that the observer has about the stimulus cannot be less than the amount of Shannon information implicit in the observer's combined binary and RT responses. In other words, the total mutual information, as measured by an experimenter, between observer responses and stimulus strength provides a lower bound for the amount of Shannon information that the observer has about the stimulus strength. Thus, each the mutual information value provided in this paper constitutes a conservative estimate of the amount of information that the observer gains about the stimulus.

### 7.1. Evaluating *I*(*x, P*)

The mutual information *I*(*x, P*) between stimulus strength *s* and the probability *P* that the observer chooses the comparison stimulus (i.e., *r* = 1) is

(23) $$I(x,P)=\int_{x}\int_{P}p(x,P)\log\frac{p(x,P)}{p(x)p(P)}\;dP\;dx$$

(24) =H(x)+H(P)−H(x,P)bits,

where *H*(*x*) and *H*(*P*) are the differential entropies of *p*(*x*) and *p*(*P*), respectively, and *H*(*x, P*) is the differential entropy of the joint distribution *p*(*x, P*). All logarithms in this paper use base 2, so information is measured in bits. Substituting *p*(*x, P*) = *p*(*P*|*x*)*p*(*x*), yields

(25) $$I(x,P)=\int_{x}p(x)\int_{P}p(P|x)\log\frac{p(P|x)}{p(P)}\;dP\;dx$$

(26) =H(P)−H(P|x)bits,

where *H*(*P*|*x*) is the differential entropy of the noise in the measurements *P*. Given Bayes' rule, *p*(*P*|*x*) = *p*(*x*|*P*)*p*(*P*)/*p*(*x*), we can recognize the mutual information as the differential entropy *H*(*P*) of the prior distribution minus the differential entropy *H*(*P*|*x*) of the posterior distribution.

We can evaluate Equation (25) by summing over discrete versions of the variables *x* and *P*. Recall that the observed proportion of responses *r* = 1 at a given stimulus strength *x*_*i*_ is *P*′_*i*_ = *n*_*i*_/*N*_*i*_, so that

(27) $$I(x,P)=\sum_{k=1}^{N_{x}}p(x_{k})\left[\sum_{i=1}^{N_{x}}p({P^{\prime }}_{i}|x_{k})\log\frac{p({P^{\prime }}_{i}|x_{k})}{p({P^{\prime }}_{i})}\right]\text{bits}.$$

We assume that the probability of stimulus values is locally uniform, so that *p*(*x*_*k*_) = 1/*N*_*k*_. In order to evaluate Equation (27), we require expressions for *p*(*P*′_*i*_|*x*_*k*_) and *p*(*P*′_*i*_).

#### 7.1.1. Evaluating the posterior *p*(*P*′_*i*_|*x*_*k*_)

Using Equation (5) across a range of *x* values, the fitted value of *P* at *x*_*k*_ is *P*_*k*_. Assuming a binomial distribution, the probability of the observed proportion *P*′_*i*_ given a fitted value *P*_*k*_ at *x*_*k*_ is

(28) $$p({P^{\prime }}_{i}|x_{k})=C_{N_{i}}^{n_{i}}\;P_{k}^{n_{i}}{\left(1-P_{k}\right)}^{N_{i}-n_{i}},$$

where *p*(*P*′_*i*_|*x*_*k*_) = *p*(*P*′_*i*_|*P*_*k*_), and *p*(*P*′_*i*_|*x*_*k*_) values are normalized to ensure that ∑_*i*_ *p*(*P*′_*i*_|*x*_*k*_) = 1.

#### 7.1.2. Evaluating the prior *p*(*P*′_*i*_)

The distribution of binary responses is binomial with a mean equal to the grand mean *P*_*G*_ of all *N*_*G*_ binary responses of an observer

(29) $$P_{G}=\frac{1}{N_{G}}\sum_{i=1}^{N_{G}}r_{i},$$

where *r*_*i*_ = 1 if and only if a response corresponds to the observer choosing the comparison stimulus. The observer's prior probability of the binary responses for the *i*th stimulus strength is therefore

(30) $$p({P^{\prime }}_{i})=C_{N_{i}}^{n_{i}}\;P_{G}^{n_{i}}{\left(1-P_{G}\right)}^{N_{i}-n_{i}},$$

where *p*(*P*′_*i*_) values are normalized to ensure that ∑_*i*_ *p*(*P*′_*i*_) = 1.

### 7.2. Evaluating *I*(*x*, τ)

Following the same line of reasoning as above, the mutual information *I*(*x*, τ) between stimulus strength and mean RT is

(31) $$I(x,\bar{\tau })=\int_{x}p(x)\int_{\bar{\tau }}p(\bar{\tau }|x)\log\frac{p(\bar{\tau }|x)}{p(\bar{\tau })}\;d\bar{\tau }\;dx$$

(32) $$=H(\bar{\tau })-H(\bar{\tau }|x)\text{bits},$$

where *H*(τ|*x*) is the differential entropy of the noise in the measurements τ.

We can evaluate Equation (31) by summing over discrete versions of the variables *x* and τ

(33) $$I(x,\bar{\tau })=\sum_{k=1}^{N_{x}}p(x_{k})\left[\sum_{i=1}^{N_{i}}p({{\bar{\tau }}^{\prime }}_{i}|x_{k})\log\frac{p({{\bar{\tau }}^{\prime }}_{i}|x_{k})}{p({{\bar{\tau }}^{\prime }}_{i})}\right]\text{bits},$$

where *p*(τ′_*i*_|*x*_*k*_) is defined by the EPRD model (Equation 8) with a fitted value τ_*k*_, so that

(34) $$p({{\bar{\tau }}^{\prime }}_{i}|x_{k})=p({{\bar{\tau }}^{\prime }}_{i}|{\bar{\tau }}_{k}(\theta_{\tau })),$$

as in Equation (18). As before, we assume that the probability of stimulus values is uniform, so that *p*(*x*_*k*_) = 1/*N*_*i*_.

#### 7.2.1. Evaluating the posterior *p*(τ′_*i*_|*x*_*k*_)

The posterior is defined in Equation (18), but is repeated here with changed subscripts for clarity

(35) $$p({{\bar{\tau }}^{\prime }}_{i}|x_{k})=\frac{1}{\sqrt{2\pi v_{\bar{\tau }k}}}\exp\left[\frac{-{\left({{\bar{\tau }}^{\prime }}_{i}-{\bar{\tau }}_{k}\right)}^{2}}{2v_{\bar{\tau }k}}\right]\text{​},$$

where *v*_τ*k*_ is defined in Equation (19), and *p*(τ′_*i*_|*x*_*k*_) values are normalized to ensure that ∑_*i*_ *p*(τ′_*i*_|*x*_*k*_) = 1.

#### 7.2.2. Evaluating the prior *p*(τ′_*i*_)

A parametric form for the observer's prior probability distribution *p*(τ) of individual RTs was estimated from the entire set of that observer's grand total of *N*_*G*_ RTs. These were fitted to an inverse Gaussian distribution to obtain a grand mean τ_*G*_ and a parameter λ_*G*_. This pdf has a variance

(36) $$v_{G}={\bar{\tau }}_{G}^{3}/\lambda_{G}.$$

At each stimulus strength *x*_*i*_, the RT mean is based on a sample of *N*_*i*_ RTs, and the central limit theorem suggests that the distribution of means is approximately Gaussian with a variance

(37) $$v_{g}=v_{G}/N_{i}.$$

Therefore, the prior probability density of each inverse Gaussian mean τ′_*i*_ is

(38) $$p({{\bar{\tau }}^{\prime }}_{i})=\frac{1}{\sqrt{2\pi v_{g}}}\exp\left[\frac{-{\left({{\bar{\tau }}^{\prime }}_{i}-{\bar{\tau }}_{G}\right)}^{2}}{2v_{g}}\right]\text{​},$$

where *p*(τ′_*i*_) values are normalized to ensure that ∑_*i*_ *p*(τ′_*i*_) = 1.

### 7.3. The shannon information of a single response

So far we have derived expressions for the Shannon information implicit in the average RT τ_*i*_ and also in the average binary response, which is summarized as the proportion *P*_*i*_ of comparison responses, for a stimulus strength *x*_*i*_. Here, we derive an expression for the Shannon information associated with a single trial; first for RTs, and then for binary responses.

As the number of trials at each stimulus strength is increased, so the variance in each mean RT decreases, and the central limit theorem ensures that the distribution of means becomes increasingly Gaussian. The mutual information between two variables (e.g., mean RT and stimulus strength) depends on the signal to noise ratio SNR

(39) $$I\leq 1/2\;\log_{2}(1+\text{SNR}),$$

where SNR is the signal variance expressed as a fraction of the noise variance in the measurement (Shannon and Weaver, 1949). If the distribution of mean RTs is Gaussian then the distribution of differences Δτ between mean RT τ and the grand mean RT (at one stimulus strength) must also be Gaussian. Because the mutual information is defined in Equation (32) to be the differential entropy of τ minus the differential entropy of the noise Δτ in τ, we can assume equality in Equation (39) (Rieke et al., 1997). In fact, we do not need to rely on the central limit theorem here, because even if the perturbing noise Δτ is not Gaussian, Shannon's Theorem 18 (Shannon and Weaver, 1949) implies equality in Equation (39), so that

(40) $$I=1/2\;\log_{2}(1+\text{SNR})\text{bits}.$$

We already have a value for the mutual information *I*(*x*, τ) from Equation (27), so we can re-arrange Equation (40) to find the SNR associated with τ

(41) $${\text{SNR}}_{\bar{\tau }}=2^{2I(x,\bar{\tau })}-1\text{bits}.$$

However, the mutual information *I*(*x*, τ) obtained from Equation (27) tells us how much average Shannon information each *mean* RT provides about stimulus strength, whereas we want to know how much average information each *individual* RT provides about stimulus strength. Because the value of SNR in Equation (41) is based on mean RTs, each of which involves *N*_*i*_ trials, the variance of the measurement noise has been reduced by a factor of *N*_*i*_ relative to the noise in the RT of a single trial (provided this noise is iid). This implies that the value of SNR for a single trial is

(42) $${\text{SNR}}_{\tau }={\text{SNR}}_{\bar{\tau }}/N_{i}$$

(43) $$=(2^{2I(x,\bar{\tau })}-1)/N_{i}\text{bits}.$$

If we substitute SNR_τ_ into Equation (40) then we obtain an estimate of the average Shannon information *I*(*x*, τ) implicit in the observer's RT in a single trial

(44) $$I(x,\tau )=\frac{1}{2}\log_{2}\left[1+\frac{\left(2^{2I(x,\bar{\tau })}-1\right)}{N_{i}}\right]\text{bits}.$$

A similar line of reasoning implies that the average Shannon information *I*(*x, r*) implicit in the observer's binary response *r* in a single trial is

(45) $$I(x,r)=\frac{1}{2}\log_{2}\left[1+\frac{\left(2^{2I(x,P)}-1\right)}{N_{i}}\right]\text{bits}.$$

In order to compare mutual information estimates for the different variables τ and *r*, the calculations for *I*(*x*, τ) and *I*(*x, r*) should be based on the same range of stimulus strengths *x*.

### 7.4. Defining the shannon increment

The mutual information between stimulus strength and (binary or RT) responses can be used to define the smallest average detectable difference in stimulus strength, which we call the *Shannon increment* (SI). We first define the effective stimulus range *x*_range_ as the range of stimulus strengths *x* associated with response probabilities between *P* = ϵ and *P* = 1 − ϵ, for some small value ϵ. Then the SI is related to the mutual information *I* by

(46) $$SI=\frac{x_{\text{range}}}{2^{I}},$$

where the value 2 is based on the assumption that information is measured in bits (i.e., using log to the base 2), and SI has the same units as stimulus strength. Because SI decreases monotonically with mutual information, it should become asymptotically closer to the true value of SI as the number of trials or stimulus strengths is increased.

A brief explanation for this definition is as follows. Consider a range of stimulus strengths *x*_range_ which give rise to “noisy” observer responses *y* = *f*(*x*), where these responses are samples from a probability density function *p*(*y*(*x*)), and where the mutual information between *x* and *y* is *I* bits. One way to interpret SI involves assuming that *p*(*y*(*x*)) is uniform. In this case, on average, knowing the value of *y* reduces the possible range of *x* values to an interval Δ*x* = *x*_range_/2^*I*^, which we can recognize as being equal to the SI.

## 8. Fat-face thin: a demonstration experiment

We used the EPRD models described above to estimate the PSE and other key parameters for a simple demonstration experiment using a human observer. On each trial, the observer was presented with a colored picture of an upright face and an inverted face (see Figure 2) on a computer screen, and was required to indicate which one appeared to be wider by pressing a left/right computer key. For half of the trials, the reference stimulus was an upright face, and the comparison stimulus was an inverted version of the same face, and these were swapped for the other half of the trials. The width of the comparison image was determined by 1 of 21 stretch factors *s* = 0.90, 0.91, …, 1.10, but the height of both stimuli was kept constant. The stimulus strength was defined to be *x* = *s* − 1, so that *x* varied between −0.1 and 0.1. For a given value of *s*_*i*_, the observer was presented with the same stimulus pair for a total of *N*_*i*_ = 20 trials. Stimuli were shown in random order, and the left/right position of reference/comparison stimuli was counterbalanced across trials.

### 8.1. Results

Each of three models defined by *L*_*P*_, *L*_τ_, and *L*_*C*_ was used to fit a psychometric and/or a chronometric function to the data from one subject, as shown in Figure 4. Maximum likelihood parameter estimation was implemented in MatLab using the Nelder–Mead simplex method. The parameter estimates for each model are summarized in Table 1.

**Table 1:** **Results for three models**.

**Model**	**PSE**	***A***	***k***	***A* × *K***	**τ_res_ (s)**	**γ**	**LLik**	**MI (bits)**
Binary *L*_*P*_	1.031 ± 0.003	NA	NA	22.32	NA	0.005	−31.13	2.68
RT *L*_τ_	1.034 ± 0.004	0.998	28.37	28.32	0.437	NA	18.7	0.87
Comb *L*_*C*_	1.032 ± 0.003	1.016	23.12	23.50	0.354	0.011	−13.10	3.18

Binary model: based only on binary response probability (Equation 12).

RT model: based only on mean RT (Equation 17).

Comb (combined model): based on binary response probability and mean RT (Equation 22).

*PSE, point of subjective equality (± indicates standard error); *A* and *k* are EPRD parameters, τ_res_ is the fixed part of RT; γ, lapse rate; LLik, log likelihood; and MI, mutual information between stimulus strength and RT or binary responses or both (see text). The final number (3.18 bits) represents *I*(*x, r*) = *2.68* plus *I*(*x*, τ) = *0.497*, computed using parameter values obtained from Equation 22*.

### 8.2. Using binary responses: model *L*_*P*_

Based on 420 binary responses, maximizing *L*_*P*_ (Equation 12) yields a psychometric function similar to that in Figure 4A, and a PSE of *s*_PSE_ = 1.031. This maximum likelihood estimate implies that an inverted face must be 3.1% wider than an upright face in order for the two faces to be perceived as the same width. Numerical estimation of the Hessian matrix of second derivatives of Equation (12) at *s*_PSE_ yields a standard error (se) of 0.003, which implies that *s*_PSE_ is significantly different from *s* = 1 (*p* < 0.001). The values of three parameters were estimated for this model, the PSE, *Ak*, and γ, and the product *Ak* is quoted in Table 1 for comparison with other works.

### 8.3. Using mean reaction times: model *L*_τ_

Each of 21 mean RTs (one per stimulus strength) was first estimated by maximizing Equation (17), based on 20 RTs per stimulus strength. Using these 21 mean RTs, *L*_τ_ (Equation 21), was maximized with respect to four parameters (PSE, *A, k*, and τ_res_) to yield a chronometric function similar to that in Figure 4B. The estimated PSE is *s*_PSE_ = 1.034 (se = 0.004, *p* < 0.001).

### 8.4. Using mean RTs and observer responses: model *L*_*C*_

Based on 42 data points (the 21 estimated mean RTs used for *L*_τ_ plus 21 corresponding binary response probabilities used for *L*_*P*_), maximizing *L*_*C*_ (Equation 22) yields the psychometric function and the chronometric function in Figures 4A,B, respectively, and a PSE of 1.032 (se = 0.003, *p* < 0.001). There are five parameters to be estimated for this model, the PSE, *A, k*, τ_res_, and γ.

### 8.5. Shannon information

The mutual information *I*(*x*, τ) between *x* and τ is the entropy in *p*(τ) and *p*(*x*) shared by the joint distribution *p*(*x*, τ). Using Equation (33), this evaluates to *I*(*x*, τ) = 2.79 bits. Using Equation (44) with *N*_*i*_ = 20, this implies that the mutual information *I*(*x*, τ) for a single RT is *I*(*x*, τ) = 0.87 bits, and is represented by the intersection of regions *X* and *Z*.

Similarly, Equation (27) can be used to estimate the mutual information between *x* and *P*, which comes to *I*(*x, P*) = 4.82 bits. Using Equation (45) with *N*_*i*_ = 20, this implies that the mutual information *I*(*x, r*) for a single binary response *r* is *I*(*x, r*) = 2.68 bits, and is represented by the intersection of regions *X* and *Y*.

We can use *I*(*x*, τ) and *I*(*x, r*) to provide lower and upper bounds on the total amount of mutual information *I*_tot_ between *x* and the combined variables (*r*, τ), which can be considered to be a vector variable. If τ and *r* provide independent information about *x* (i.e., if *a* = 0 in Figure 1) then the maximum value of *I*_tot_ is

(47) $$\max(I_{\text{tot}})=I(x,\tau )+I(x,r)$$

(48) =0.87+2.68

(49)      = 3.55 bits.

However, if all of the information *I*(*x*, τ) provided by τ about *x* is the same as part of the information provided by *r* about *x* (i.e., if *c* = 0 in Figure 1) then *I*_tot_ cannot be less than *I*(*x, r*). To take account of the possibility that all of the information *I*(*x, r*) provided by *r* about *x* is the same as part of the information provided by τ about *x*, we can write

(50) $$\min(I_{\text{tot}})=\max(I(x,\tau ),I(x,r))$$

(51) =max(0.87,2.68)

(52) =2.68 bits.

Thus, on average, each trial provides the observer with between 2.68 and 3.55 bits.

### 8.6. Shannon increment

Using a conservative estimate of mutual information of *I* = 2.68 bits suggests that the observer can discriminate differences between the reference and comparison stimulus with an average resolution of about one part in 6.39 (= 2^2.68^) of the effective range *x*_range_ of stimulus strengths. Note that the range of scaling values used *s*_range_ = 0.2 (i.e., 0.9 … 1.1) equals the range of stimulus strengths *x*_range_ = 0.2 (i.e., −0.1 … 0.1). Therefore, the SI for the width scaling factor is

(53) $$SI=x_{\text{range}}/2^{I}$$

(54)     = 0.2/6.39

(55)     = 0.031,

where we have assumed ϵ = 0 here. Thus, on average, the smallest change in scaling factor (between reference and comparison stimulus) detectable by the observer is SI = 0.031.

## 9. Discussion

We have shown how the PRD model from Palmer et al. (2005) can be extended to make use of individual RTs, which can be combined with binary observer responses to estimate key psychophysical parameters in a 2AFC design.

A key feature of diffusion-based models is that they treat each RT as the end-point of an accumulation of evidence. If we take this type of evidence-accumulation process seriously then it makes sense to model the distribution of RT values as an inverse Gaussian distribution (for reasons described in section 5).

A striking result is the difference between the log likelihoods associated with the binary response model and the RT model, despite the fact that the binary response model has fewer free parameters than the RT model, and that both models provide similar PSE estimates which (based on their sems, not shown) are not significantly different. These log likelihood values suggest that the EPRD model provides a better fit to the RT data than it does to the binary response data. This difference in likelihoods suggests that the parameter estimates obtained using the combined RT and response data is dominated by the binary data likelihood term.

Self-evidently, both the RT and binary responses of an observer depend on the stimulus strength *x*. However, in general, it is not known if RT or binary response data provide more Shannon information about the value of *x*. More importantly, and more subtley, it is not known if they provide the same information about *x*, or if they merely provide the same *amount* of information about *x* (see Figure 1).

We can gain some insight into the nature of this problem by considering the proportion of the differential entropy in stimulus values accounted for by the corresponding differential entropy in observer responses. At one extreme, if an observer is told to respond as quickly as possible then the RTs should provide relatively large amounts of mutual information regarding stimulus strength, whereas the binary responses carry relatively little mutual information (because speeded responses tend to be inaccurate Hanks et al., 2011). In this case, the RT entropy at a given stimulus strength will be relatively small, because RTs will be tightly coupled to the stimulus strength, whereas the binary response entropy at a given stimulus strength will be relatively large (because these responses are inaccurate, and therefore not tightly coupled to the stimulus strength). However, when considered across different stimulus strengths, the tight coupling between RT and stimulus strength will give rise to a relatively large RT entropy, and most of this entropy will be shared with stimulus strength entropy (which defines a large mutual information between RT and stimulus strength). In contrast, these fast, inaccurate responses across stimulus strengths will be associated with a relatively small range of response probability values (e.g., *P* ≈ 0.5), which will therefore have a relatively small entropy, most of which is not shared with the stimulus strength entropy (which defines a small mutual information between binary responses and stimulus strength). In summary, fast responses should yield high entropy RT values, which share a large proportion of their entropy with the stimulus strength, combined with low entropy *P* values which share a small proportion of their entropy with the stimulus strength. At the other extreme, if an observer is told to be as accurate as possible then this should yield high entropy *P* values which share a large proportion of their entropy with the stimulus strength, combined with low entropy RT values which share a small proportion of their entropy with the stimulus strength. In summary, the entropy in stimulus strength can be shared with entropy in both accuracy (*P*) and speed (RT). However, as there is probably only a finite amount of such shared entropy (mutual information) available, we predict that it can be realized experimentally as maximum speed or maximum accuracy, but not both.

The scenario considered above can be represented geometrically, as in Figure 1. If we compare the mutual information between τ and *x* with the mutual information between *r* and *x* then it is possible that they have the same magnitude [e.g., (*a* + *c*) = (*a* + *b*), as in Figure 1]. However, the fact that both τ and *x* have the same *amount* of mutual information (i.e., they account for the same amount of entropy in *x*) does not imply that they account for the same *entropy* in *x*. Formally, the fact that (*a* + *c*) = (*a* + *b*) does not imply that (*a* + *c*) ≡ (*a* + *b*). This matters because, even if *I*(*x*, τ) = *I*(*x, r*), we could not conclude that *I*(*x*, τ) ≡ *I*(*x, r*), and so we could not conclude that τ and *r* provide mutually redundant information. Thus, we cannot dismiss τ simply because *r* accounts for more entropy in *x* than τ does (or vice versa). Indeed, this is precisely the situation that we have in the results reported here, and provides reasonable grounds for making use of both RT and binary response data in general.

Unfortunately, we have been unable to derive an expression for the total mutual information between the joint variables (RT and binary responses) and stimulus strength *I*(τ, *P*; *x*′) (i.e., the area [*a* + *b* + *c*] in Figure 1), although it may be possible to do so using Equation (10) [where the entropy of the difference between *P* and τ is *H*(τ, *P*|*x*′)]. The precise effect of the instructions given to observers on mutual information, and the proposed invariance of the total mutual information with respect to instructions, clearly require further research (Soukoreff and MacKenzie, 2009).

The Shannon increment (SI) is similar in spirit to the more conventional just noticeable difference (JND). However, the JND has an arbitrary value, and (despite its name) there is no reason to suppose that a JND is indeed just noticeable. The SI is monotonically related to the average amount of Shannon information an observer gains regarding a single presentation of a stimulus, and is a measure of the perceptual resolution with which a parameter is represented by the observer.

## 10. Conclusion

We have presented an extended proportional-rate diffusion model, which takes account of both individual RTs and binary responses for maximum likelihood estimation of key psychophysical parameters (e.g., PSE, slope) of the psychometric and chronometric functions. The fact that these psychophysical parameters have similar estimated values when computed independently for two models based on RTs alone or on binary responses alone provides support for the underlying physical basis of this class of diffusion models.

An information-theoretic analysis was used to estimate the average amount of Shannon information that each RT provided about the stimulus value, and also the average amount of Shannon information that each binary response provided about the stimulus value. This analysis provides bounds for the average amount of Shannon information that the observer gains about the stimulus value from one presentation, which was found to be between 2.68 and 3.55 bits/trial for the experiment used here.

### Conflict of interest statement

The author declares that the research was conducted in the absence of any commercial or financial relationships that could be construed as a potential conflict of interest.

## Footnotes

^1^Notice that, if the lapse rate is γ = 0.01 then the upper and lower bounds are 0.995 and 0.005, respectively, because half of the observer's guesses will be correct, on average.

^2^For reference, the Wald distribution is the distribution of first passage times of a biased Brownian process, and is qualitatively similar to the log-normal distribution, which is often used to model RT.

## Appendix

### Mathematical symbols and abbreviations

*A* an EPRD model parameter which is the amount of evidence required to trigger a response.
*comparison stimulus response*: a response indicating the comparison stimulus was chosen.
*EPRD*: extended proportional-rate diffusion model.
*SI*: Shannon increment, the smallest detectable change in a stimulus.
γ EPRD lapse rate parameter.
*i* index over stimulus strength *x*, with range *i* = 1, …, *N*_*x*_.
*j* index over trials at one stimulus strength *x*_*i*_, with range *j* = 1, …, *N*_*i*_.
*k* index over stimulus strength, with range *k* = 1, …, *N*_*x*_.
*K* is a measure of sensitivity to changes in *x* in the EPRD model.
*N*_*i*_ number of trials at stimulus strength *x*_*i*_.
*N*_*x*_ number of different stimulus strengths.
*PSE*: point of subjective equality.
*P*_*i*_ proportion of comparison stimulus responses at stimulus strength *x*_*i*_, predicted by EPRD model.
*P*′_*i*_ MLE mean, equal to observed proportion of comparison responses at stimulus strength *x*_*i*_.
*r* binary observer response (e.g., observer chooses comparison or reference stimulus).
*s*_*C*_ variable stimulus value of the comparison stimulus.
*s*_*R*_ fixed stimulus value of the reference stimulus.
*s*_PSE_ value of the comparison stimulus which the observer perceives as being the same as the reference stimulus.
τ′_*i*_ MLE mean of inverse Gaussian RT at stimulus strength *x*_*i*_.
τ_*i*_ mean RT at stimulus strength *x*_*i*_, as predicted by EPRD model.
τ_dec,*i*_ mean decision RT at stimulus strength *x*_*i*_, as predicted by EPRD model.
τ_res_ mean residual RT (assumed the same at all stimulus strengths), as predicted by EPRD model, where τ_res_ = τ_dec,*i*_ − τ_*i*_.
θ_τ_ = (*s*_PSE_, *A, K*, γ, τ_res_), five parameters for the RT component of the EPRD model.
θ_*P*_ = (*s*_PSE_, *A K*, γ), three parameters for the binary response component of the EPRD model.
*v*_τ, *i*_ variance in mean RT.
*x*_*i*_ stimulus strength.
*x*′_*i*_ perceived strength of stimulus with strength *x*_*i*_.
